# Health-related quality of life of patients with HIV/AIDS at a tertiary care teaching hospital in Ethiopia

**DOI:** 10.1186/s12955-021-01670-7

**Published:** 2021-01-19

**Authors:** Yared Belete Belay, Eskinder Eshetu Ali, Beate Sander, Gebremedhin Beedemariam Gebretekle

**Affiliations:** 1 School of Pharmacy, College of Health Sciences, Mekelle University, Mek’ele, Ethiopia; 2 Department of Pharmaceutics and Social Pharmacy, School of Pharmacy, College of Health Sciences, Addis Ababa University, Addis Ababa, Ethiopia; 3 Toronto Health Economics and Technology Assessment (THETA) Collaborative, University Health Network, Toronto, ON Canada; 4 Institute of Health Policy, Management and Evaluation (IHPME), University of Toronto, Toronto, ON Canada; 5 Public Health Ontario, Toronto, ON Canada; 6 ICES, Toronto, ON Canada

**Keywords:** EQ-5D-5L, HIV/AIDS, Quality of life, Utility, EuroQol

## Abstract

**Background:**

Patients’ health-related quality of life (HRQoL) and health state utility values are critical inputs in the clinical and economic evaluation of treatments for human immunodeficiency virus (HIV)/acquired immunodeficiency syndrome (AIDS). However, information on health state utility values is lacking in the context of Ethiopia. Here, we aimed to assess HRQoL and determine health state utility values and factors that influence the values among HIV/AIDS patients in Ethiopia.

**Methods:**

A cross-sectional study was conducted among 511 HIV/AIDS patients at Tikur Anbessa Specialized Hospital in Ethiopia. Patients aged 18 years or older were eligible for the interview and those who were mentally unstable and with hearing impairment were excluded from the study. We performed face-to-face interviews using EuroQol-5 Dimensions-5 Levels (EQ-5D-5L) in combination with EuroQol-Visual Analog Scales (EQ-VAS). Level-specific disutility coefficients obtained from the general population were used for computing utility values. Patients’ health profiles were described using percentages and different statistical analysis were conducted to determine factors associated with the EQ-5D index and EQ-VAS scores.

**Results:**

A total of 511 patients participated in the study. A higher proportion of patients reported slight or more severe problems on the anxiety/depression (55.2%) and pain/discomfort (51.3%) dimensions. The overall median utility value of HIV/AIDS patients was 0.94 (IQR = 0.87, 1) from the EQ-5D index and 80% (IQR = 70%, 90%) from the EQ-VAS scores. Demographic characteristics including age, occupational status, and household monthly income significantly affected patient’s utility values. Moreover, statistically significant (*p* < 0.001) differences were seen between the EQ-5D index values of patients with different CD4 count intervals. Furthermore, number of medicines that the patients were taking at the time of the study and comorbidities were significantly associated with the EQ-5D utility index and EQ-VAS score, *p* < 0.001.

**Conclusions:**

The anxiety/depression and pain/ discomfort dimensions were identified to have critical influence in reducing the HRQoL of adult HIV/AIDS patients in the context of Ethiopia. The study is also the first to use the EQ-5D-5L tool to identify health state utility values for Ethiopian adult HIV/AIDS patients. Future economic evaluations of HIV/AIDS interventions are encouraged to employ the identified utility values.

## Introduction

Human immunodeficiency virus (HIV)/acquired immunodeficiency syndrome (AIDS) is a major issue of public health relevance globally. The health and economic burden of the disease is even more daunting in low-income countries, which include most African nations [1–3]. Ethiopia is not an exception and HIV/AIDS is a significant health concern in the country. With a one percent annual prevalence, the disease accounts for 19% of Ethiopia’s national health expenditure, despite significant improvements as a result of increased access to antiretroviral treatment (ART) [4, 5].

Although significant progress has been made in drug discovery for the treatment of HIV/AIDS, the currently available medicines fall short of curing the disease. The lack of curative therapy impacts patients’ health-related quality of life (HRQoL) and treatment outcomes [6, 7]. Hence, health-related disability and disfunction should be assessed using HRQoL. The health state utility value which is a numerical description of HRQoL can be used to express HRQoL and it incorporates the preference of people. Both the health state utility and HRQoL helps to assess the need for and outcome of interventions from the experience of patients. Health state utility value is the preferred parameter used in health technology assessment [8, 9]. Several tools have been developed and validated for measuring HRQoL in patients including WHOQOL-HIV instrument and EQ-5D [10–12]. The capacity of these tools range from simple measurement of symptom burdens (such as the World Health Organization Quality of Life in HIV (WHOQOL-HIV) instrument) to the quantification of health state utility values. In this regard, the EuroQol-5 dimensions (EQ-5D) tool is significant because of its ability to generate utility scores, which are critical in full-fledged health economic evaluations involving cost utility analysis [10, 13]. In such analyses, the utility is used as a good metric for comparison of quality-adjusted life-year between alternative interventions [14–16].

In Ethiopia, several studies have assessed HRQoL of HIV/AIDS patients using the WHOQOL-HIV instrument [17–20]. However, there is a paucity of studies that employ tools such as the EQ-5D that incorporate societal preference and provide effective means to assess impact of the disease across different health states [14]. As a result, health care decision-makers in Ethiopia still lack well-established utility values for HIV/AIDS patients based on different health states of the disease. To this effect, the current study aimed to assess HRQoL and determine health state utility values and factors that influence the values among HIV/AIDS patients in Ethiopia.

## Methods

### Study design and setting

An institution-based cross-sectional study was conducted from April to July 2019 at Tikur Anbessa Specialized Hospital (TASH), the largest tertiary teaching hospital (affiliated with Addis Ababa University) in Ethiopia. The hospital has 1,204 health professionals and 800 beds serving more than 500,000 patients per year. The ART clinic pharmacy is one of the 12 pharmacy departments within the hospital and approximately 4,000 patients are served annually in this pharmacy.

### Recruitment of participants

All patients visiting the ART pharmacy at TASH were considered source population while patients visiting the ART pharmacy of the hospital during the data collection period were the study population. Participants had to be 18 years or older to be eligible for the interview. In contrast, patients with incomplete medical chart records (absence of CD4 data), those who were mentally unstable and with hearing impairment were excluded from the study.

The total number of participants was estimated based on a single proportion formula [21], considering a Z-value of 1.96 with a 95% level of confidence and 4% of a margin of error. The proportion (P) for sample size estimation was taken from a previous study which assessed the quality of life of HIV/AIDS patients at university hospital in Ethiopia and reported that ~ 33% of patients rated their overall perceived HRQoL as good [19]. According to TASH’s ART pharmacy electronic dispensing tool, the total number of active ART users was 3,828 in March 2019. With a 5% non-response rate, the final sample size was estimated to be 511.

### Data collection instrument

In this study, the Amharic versions of the 5-level EQ-5D (EQ-5D-5L) and the EuroQol-visual analog scale (EQ-VAS) were used to evaluate HRQoL and generate utility scores. EQ-5D is a generic, preference-based and multi-attribute utility evaluation instrument developed by the EuroQol group. It is a widely recommended tool to generate input data for economic evaluation [13]. The tool was originally developed to include three levels of severity in five dimensions (EQ-5D-3L). Since 2009, the EuroQol group introduced the 5-level EQ-5D version, which is shown to have better sensitivity and reduced ceiling effect in comparison to EQ-5D-3L. EQ-5D-5L is designed to include mobility, self-care, usual activities, pain/discomfort, and anxiety/depression dimensions. Each dimension is then measured on five levels ranging from no problem to an extremely severe problem or unable to perform any activities-VAS was used to record patient’s self-rated health status on a 20-cm vertical scale with endpoints 0 (‘the worst health you can imagine’) and 100 (‘the best health you can imagine’) [15, 22]. Prior studies involving samples from the general population showed that the Amharic versions of EQ-5D-5L and EQ-VAS instruments are feasible and culturally acceptable in the context of Ethiopia [23].

In addition to EQ-5D-5L and EQ-VAS, the data collection tool contained questions related to patients’ socio-demographic characteristics and checklist for recording clinical characteristics from the patient medical charts. Except for the checklist, all the questions were in the Amharic language (the working language of the country), to ensure understandability by patients. Since the information in the patient chart was documented in English, using the English version of the checklist was suitable to avoid potential confusion during data collection.

### Data collection procedure and quality assurance

Data collection was done by one postgraduate pharmacy student and two pharmacy staff working in the ART pharmacy who took a half-day training on the proper conduct of the study. Face-to-face interviews were carried out to collect data on socio-demographic, EQ-5D-5L and EQ-VAS questions. Information related to clinical characteristics were extracted from the medical records after completion of the interview with patients. All the collected data were checked for their completeness, accuracy and consistency by the first author (YBB) on the site of data collection. Prior to analysis, data cleaning was done by observing the descriptive statistics output.

### Data analysis

The utility score was calculated using Microsoft excel. Patient health profiles from EQ-5D-5L were converted into a single index (utility) based on the preference of the general population of Ethiopia. EQ-5D-5L index and EQ-VAS scores were used to describe the HRQoL of the patients. Coefficients that represented level-specific disutility values obtained from the general population using a hybrid regression model were used for computing utility value. Four dummies were created for each dimension by considering level one as a reference to represent a decrease in utility in moving from one level to the next higher level (e.g. moving from MO1 to MO2 resulted in a utility decrement of 0.0337341). The 20 parameters reported from the general population for all the five health domains [23] were used to compute utility score (Eq. 1).

1 $$\begin{aligned} {\text{Utility value}} & = {1} - ({\text{mo2}}*0.0{337341} + {\text{mo3}}*0.0{644715} + {\text{mo4}}*0.{2276493} + {\text{mo5}}*0.{3598963}) \\ & \quad + ({\text{sc2}}*0.0{235125} + {\text{sc3}}*0.0{394815} + {\text{sc4}}*0.{1419238} + {\text{sc5}}*0.{2223553}) \\ & \quad + ({\text{ua2}}*0.0{323}0{13} + {\text{ua3}}*0.0{482993} + {\text{ua4}}*0.{1573934} + {\text{ua5}}*0.{2721253}) \\ & \quad + {\text{(pd2}}*0.0{36}0{8}0{8} + {\text{pd3}}*0.0{515949} + {\text{pd4}}*0.{27}0{3189} + {\text{pd5}}*0.{4}0{63984)} \\ & \quad + {\text{(ad2}}*0.0{258862} + {\text{ad3}}*0.0{848133} + {\text{ad4}}*0.{2987388} + {\text{ad5}}*0.{4577938)} \\ \end{aligned}$$

*mo = mobility; *sc = self-care; *ua = usual activity; *pd = pain/discomfort; *ad = Anxiety/depression.

**Equation ****1**. Equation for computing utility score.

Statistical analysis was performed using IBM SPSS Statistics for Windows, Version 23.0. (IBM Corp., Armonk, NY, USA). Descriptive statistics including frequency and percentage were used to describe the health profile of the study population. Kolmogorov–Smirnov, Shapiro–Wilk test (*p* < 0.001) and visual inspection of histogram revealed that the distribution of EQ-5D index values and EQ-VAS scores were skewed and kurtotic. Therefore, we used a non-parametric test to compare the utility value across different groups. Median and interquartile range (IQR) were used to characterize the included samples with respect to EQ-5D and EQ-VAS output. Mann–Whitney U test was used to assess significance of difference of utilities for variables with two categories while Kruskal–Wallis test was used for comparison of utility value among variables with more than two groups. The mean rank was used to compare the difference across different groups and *p-value* < 0.05 was considered as a cut-point for determining statistical significance.

## Results

### Participants’ demographic and clinical characteristics

This study enrolled a total of 511 patients with wide-ranging demographic and clinical characteristics. The mean age of the participants was 42 ± 11 years (ranged: 18 to 80 years). A total of 325 (63.6%) of the participants were within the age range of 30–49 years; 395 (77.3%) never drunk alcohol and 496 (97.1%) never smoked cigarettes (Table 1). The majority of the participants (407, 80%) had a CD4 value of above 200 cells/mm^3^ and few (22, 4.3%) had CD4 count below 100 cells/mm^3^. In contrast, 440 (86%) participants had a viral load below the limit of detection (50 copies/ml). With 297 (58.1%) of patients taking it, TDF/3TC/EFV was found to be the most frequently prescribed ART regimen (Table 2).

**Table 1:** Socio-demographic characteristics and reported health problem of HIV/AIDS patients, TASH, Ethiopia, 2019 (n = 511)

Patient characteristics	Total number of participant n (%)	Reported health problem in EQ-5D health dimensions				
		Mobility n (%)	Self-care n (%)	Usual activity n (%)	Pain/Discomfort n (%)	Anxiety/Depression n (%)
*Gender*						
Male	202 (39.5)	33 (16.3)	20 (9.9)	41 (20.3)	99 (49.0)	112 (55.4)
Female	309 (60.5)	52 (16.8)	21 (6.8)	58 (18.8)	163 (58.2)	170 (55.0)
*Age (in years)*						
18–29	63 (12.3)	4 (6.3)	3 (4.8)	11 (17.5)	38 (60.3)	45 (71.4)
30–39	141 (27.6)	21 (14.9)	7 (5.0)	20 (14.2)	63 (44.7)	70 (49.6)
40–49	184 (36)	28 (15.2)	13 (7.1)	34(18.5)	94 (51.1)	94 (51.1)
50–59	77 (15.1)	19 (24.7)	9 (11.7)	17 (22.1)	34 (44.2)	41 (53.2)
> 60	46 (9.0)	13 (28.3)	9 (19.6)	17 (37.0)	33 (71.7)	32 (69.6)
*Marital status*						
Unmarried	121 (23.7)	17(14.0)	5 (4.1)	24 (19.8)	63 (52.1)	73 (60.3)
Married	278 (54.4)	233 (83.8)	25 (9.0)	48 (17.3)	132 (47.5)	141 (50.7)
Divorced	47 (9.2)	6 (12.8)	2 (4.3)	11 (23,4)	26 (55.3)	24 (51.1)
Widowed	65 (12.7)	17 (26.2)	9 (13.8)	16 (24.6)	41 (63.1)	44 (67.7)
*Education*						
No formal education	61 (12.0)	9 (14.8)	5 (8.2)	11 (18.0)	39 (63.9)	36 (59.0)
Grade(1–4)	88 (17.2)	14 (15.9)	9 (10.2)	23 (26.1)	44 (50.0)	53 (60.2)
Grade (5–8)	133 (26.0)	23 (17.3)	17(12.8)	30 (22.6)	70 (52.6)	73 (54.9)
High school	132 (25.8)	29 (22.0)	5 (3.8)	20 (15.2)	66 (50.0)	71 (53.8)
TVET/Diploma	61 (12.0)	6 (9.8)	4 (6.6)	10 (16.4)	29 (47.5)	35 (57.4)
1st degree and above	36 (7.0)	4 (11.1)	1 (2.8)	5 (13.9)	14 (38.9)	14 (38.9)
*Occupation*						
Employed	92 (18.1)	17 (18.5)	8 (8.7)	11 (12.0)	43 (46.7)	45 (48.9)
Private business	265 (52.1)	33 (12.5)	14 (5.3)	49 (18.5)	132 (49.8)	142 (53.6)
Pensioner	13 (2.6)	5 (38.5)	4 (30.8)	7 (53.8)	11 (84.6)	11 (84.6)
Student	17 (3.3)	1 (5.9)	–	2 (11.8)	11 (64.7)	13 (76.5)
Housewife	97 (19.1)	19 (19.6)	10 (10.3)	19 (19.6)	46 (47.4)	54 (55.7)
Others*	25 (4.9)	9 (36.0)	5(20.0)	10 (40.0)	17 (68.0)	15 (60.0)
*Income*						
< 1700 ETB	164 (33.7)	34 (20.7)	15 (9.1)	38 (23.2)	92 (56.1)	95 (57.9)
1700–5000 ETB	222 (45.6)	33 (14.9)	15 (6.8)	44 (19.8)	123 (55.4)	135 (60.8)
> 5000 ETB	101 (20.7)	11 (10.9)	8 (7.9)	11 (10.9)	38 (37.6)	42 (41.6)
*Smoking habit*						
Daily	2 (0.4)	–	–	–	–	1 (50.0)
Occasionally	13 (2.5)	1 (7.7)	–	4 (30.8)	8 (61.5)	9 (69.2)
Not at all	496 (97.1)	84 (16.9)	41 (8.3)	95 (19.2)	254 (51.2)	272 (54.8)
*Alcohol taking habit*						
Never	395 (77.3)	71(18)	35 (8.9)	80 (20.3)	202 (51.1)	211 (53.4)
Monthly or less	87 (17)	10 (11.5)	4 (4.6)	14 (16.1)	47 (54)	53 (60.9)
2–4 times a month	24 (4.7)	2 (8.3)	1 (4.2)	3 (12.5)	11 (45.8)	15 (62.5)
2–3 times a week	5 (1.0)	2 (40.0)	1 (20.0)	2 (40.0)	2 (40.0)	3 (60.0)
*Disclosure*						
Yes	502 (98.2)	80 (15.9)	40 (8.0)	96 (19.1)	256 (51.0)	277 (55.2)
No	9 (1.8)	5 (55.6)	1 (11.1)	3 (33.3)	6 (66.7)	5 (55.6)

TVET, Technical and Vocational Education and Training

*Others: Seasonal and unemployed

**Table 2:** Clinical characteristics and reported health problem of patients with HIV/AIDS, TASH, Ethiopia, 2019 (n = 511)

Patient characteristics	Number of participant n (%)	Reported health problem in EQ-5D health dimensions				
		Mobility n (%)	Self-care n (%)	Usual activity n (%)	Pain/Discomfort n (%)	Anxiety/Depression n (%)
*CD4 (Cells/mm*^*3*^*)*						
> 500	138 (27.0)	15 (10.9)	1 (0.7)	11 (8.0)	40 (29.0)	39 (28.3)
350–500	129 (25.2)	22 (17.1)	12 (9.3)	24 (18.6)	65 (50.4)	72(55.8)
200–350	142 (27.8)	21 (14.8)	14 (9.9)	31 (21.8)	84 (59.2)	95 (66.9)
100–200	80 (15.7)	22 (27.5)	10 (12.5)	26 (32.5)	59 (73.7)	60 (75.0)
< 100	22 (4.3)	5 (22.7)	4 (18.2)	7 (31.8)	14 (63.6)	16 (72.7)
*WHO clinical stages (at diagnosis)*						
I	67 (13.3)	16 (23.9)	3 (4.5)	10 (14.9)	40 (59.7)	42 (62.7)
II	77 (15.2)	11 (14.3)	6 (7.8)	15 (19.5)	40 (51.9)	40 (51.9)
III	190 (37.6)	28 (14.7)	16 (8.4)	32 (16.8)	83 (43.7)	95 (50.0)
IV	171 (33.9)	27 (15.8)	14 (8.2)	40 (23.4)	97 (56.7)	102 (59.6)
*Viral load (Copies/mL)*						
< 50 copies/ml	440 (86.1)	70 (15.9)	33 (7.5)	81 (18.4)	217 (49.3)	236 (53.6)
51–1000 copies/ml	17 (3.3)	3 (17.6)	2 (11.8)	2 (11.8)	9 (52.9)	10 (58.8)
> 1000 copies/ml	54 (10.6)	12 (22.2)	6 (11.1)	16 (29.6)	36 (66.7)	36 (66.7)
*Treatment regimen*						
AZT/3TC/EFV	30 (5.9)	5 (16.7)	1 (3.3)	4 (13.3)	10 (33.30	13 (43.3)
AZT/3TC/NVP	28 (5.5)	4 (14.3)	1 (3.6)	3 (10.7)	14 (50)	13 (46.4)
TDF/3TC/EFV	297 (58.1)	49 (16.5)	22 (7.4)	51 (17.2)	146 (49.2)	161 (54.2)
TDF/3TC/NVP	37 (7.2)	4 (10.8)	4 (10.8)	10 (27.0)	19 (51.4)	19 (51.4)
ABC + 3TC + EFV	40 (7.8)	9 (22.5)	5 (12.5)	8 (20.0)	28 (70.0)	27 (67.5)
TDF + 3TC + ATV/r	41 (8.0)	7 (10.8)	3 (7.3)	12 (29.3)	27 (65.9)	29 (70.7)
Others*	38 (7.4)	7 (18.4)	5 (13.2)	11 (28.9)	18 (47.4)	20 (52.6)
*Number of medicine*						
1	208 (40.7)	25 (12)	12 (5.8)	25 (12)	88 (42.3)	93 (44.7)
2	150 (29.4)	26 (17.3)	11 (7.3)	31 (20.7)	84 (56.0)	90 (60.0)
3	91 (17.8)	17 (18.7)	8 (8.8)	21 (23.1)	49 (53.8)	53 (58.2)
4	43 (8.4)	10 (23.3)	3 (7)	12 (27.9)	27 (62.8)	30 (69.8)
> 4	19 (3.7)	7 (36.8)	7 (36.8)	10 (52.6)	14 (73.7)	16 (84.2)
*Duration of treatment*						
< 1 year	4 (0.8)	–	–	–	4(100)	1 (25)
1–5 years	120 (23.5)	19 (15.8)	8 (6.7)	27 (22.5)	74 (61.7)	77 (64.2)
> 5 years	386 (75.7)	66 (17.1)	33 (8.5)	72 (18.7)	183 (47.4)	203 (52.6)
*Adherence*						
Good	466 (91.5)	77 (16.5)	38 (8.2)	90 (19.3)	236 (50.6)	252 (54.1)
Fair	13 (2.6)	3 (13.1)	1 (7.7)	–	7 (53.8)	9 (69.2)
Poor	30 (5.9)	5 (16.7)	2 (6.7)	9 (30)	17 (56.7)	20 (66.7)
*History of Rx failure*						
Yes	94 (18.4)	17 (18.1)	9 (9.6)	23 (24.5)	60 (63.8)	62 (66)
No	417 (81.6)	68 (16.3)	32 (7.7)	76 (18.2)	202 (48.4)	220 (52.8)
*Comorbidities*						
No	349 (68.3)	47 (13.5)	21 (6)	53 (15.2)	156 (44.7)	175 (50.1)
OIs	41 (8)	5 (12.2)	2 (4.9)	13 (31.7)	25 (61)	28 (68.3)
Non-OIs	121 (23.7)	33 (27.3)	18 (14.9)	33 (27.30	81 (66.9)	79 (65.3)

Rx, treatment; NVP, Nevirapine; ABC, Abacavir; OIs, Opportunistic infections

*Others: ABC + 3TC + NVP, TDF + 3TC + LPV/r and AZT + 3TC + LPV/r

### Participants’ health profile

Overall, 92%, 83.4% and 80.6% of the participants reported “no problem” in the self-care, mobility and usual activity domains, respectively. In contrast, 55.2% and 51.3% of participants reported at least slight problems on the anxiety/depression and pain/discomfort dimensions respectively (Table 3).

**Table 3:** Frequency of self-reported health profile of HIV/AIDS patients, TASH, Ethiopia, 2019 (n = 511)

Health profile	Mobility n (%)	Self-care n (%)		Usual activity n (%)	Pain/Discomfort n (%)	Anxiety/Depression n (%)
No problem	426 (83.4)	470 (92)		412 (80.7)	249 (48.7)	229 (44.8)
Slight problem	48 (9.4)	33 (6.4)		71 (13.9)	143 (28)	151 (29.5)
Moderate problem	22 (4.3)	4 (0.8)		22 (4.2)	87 (17)	103 (20.2)
Severe problem	15 (2.9)	4 (0.8)		6 (1.2)	32 (6.3)	27 (5.3)
Extreme problem	0	0		0	0	1 (0.2)

With respect to participants’ health profile based on their clinical and socio-demographic characteristics, 163 (58.2%) of female participants reported at least a slight problem on the pain/discomfort dimension. A nearly similar proportion of male (112, 55.4%) and female (170, 55%) participants reported at least a slight problem on the anxiety/depression dimension. Higher proportion of participants (45, 71.4%) from the younger age group (18–29 years) reported having at least a slight problem on the anxiety/depression dimension. In contrast, 33 (71.7%) of participants who were older than 60 years claimed having at least a slight problem on pain/discomfort dimension (Table 1). The number of participants with CD4 count of > 500 cells/mm^3^ who reported at least a slight problem on the anxiety/depression dimension was found to be 39 (28.3%). In contrast, 60 (75%) of participants with CD4 count of 100–200 cells/mm^3^ reported having a slight or more problems on the anxiety/depression dimension. Similarly, 16 (84.2%) and 14 (73.7%) of participants taking more than four medicines at a time reported a slight or more problems on the anxiety/depression and pain/discomfort dimensions, respectively (Table 2).

### Utility values and factors influencing patients’ utility values

The overall median utility value of HIV/AIDS patients was found to be 0.94 (IQR = 0.87, 1) and 80% (IQR = 70%, 90%) from the EQ-5D index and EQ-VAS result, respectively. The median utility values were found to be higher for participants in some socio-demographic and clinical characteristics categories. For instance, the median utility values were 0.96 (IQR = 0.88, 1) and 0.94 (IQR = 0.77–0.97) for participants in the age range of 30–39 years and those with a monthly household income of greater than 5000 ETB, respectively. There were significant differences in the EQ-5D utility index among different age groups, *p* = 0.004, with a mean rank utility index of 232, 282, 259, 260 and 192 for the age range of 18–29, 30–29, 40–49, 50–59 and > 60, respectively. The difference in monthly household income showed significant difference in utility index, *p* < 0.001, with mean rank utility value of 225 for < 1700 ETB, 233 for 1700–5000 ETB and 299 for > 5000 ETB. The Kruskal–Wallis test depicted that age, occupational status, and household monthly income significantly associated with participants' EQ-VAS scores with *p* < 0.001, 0.008 and 0.007, respectively (Table 4).

**Table 4:** Socio-demographic factor associated with EQ-5D and EQ-VAS score of patients with HIV/AIDS, TASH, Ethiopia, 2019 (n = 511)

Patient characteristics	EQ-5D			EQ-VAS		
	Median [IQR]	Mean rank	*p* value	Median [IQR]	Mean rank	*p* value
*Gender*						
Male	0.96 [0.86–1]	261	0.54	85 [70,90]	252	0.65
Female	0.94 [0.87–1]	253		80 [70–95]	258	
*Age*						
18–29	0.94 [0.84–0.97]	232	0.004*	80 [60–90]	217	< 0.001*
30–39	0.96 [0.88–1]	282		85 [70–95]	284	
40–49	0.94 [0.88–1]	259		85 [70–95]	271	
50–59	0.94 [0.86–1]	260		80 [70–90]	250	
> 60	0.89 [0.75–0.97]	192		65 [50–85.5]	177	
*Marital status*						
Unmarried	0.96 [0.85–1]	253	0.11	80 [70–95]	251	0.26
Married	0.96 [0.88–1]	268		85 [70–95]	268	
Divorced	0.94 [0.88–0.97]	250		80 [65–90]	243	
Widowed	0.92 [0.86–0.97]	216		80 [60–90]	224	
*Education*						
No formal education	0.94 [0.86–0.97]	233	0.27	80 [65–90]	234	0.24
Grade(1–4)	0.94 [0.86–1]	255		80 [64.75–91.25]	245	
Grade (5–8)	0.94 [0.88–1]	250		80 [70–90]	246	
High school	0.94 [0.86–1]	252		80 [70–95]	262	
TVET/diploma	0.96 [0.88–1]	276		85 [70–95]	280	
1st degree and above	0.97 [0.89–1]	301		90 [78.75–95]	293	
*Occupational status*						
Employed	0.97 [0.88–1]	280	0.001*	85 [75–93.75]	280	0.008*
Private business	0.94 [0.88–1]	261		85 [70–90]	255	
Pensioner	0.85 [0.83–93]	146		70 [45–80]	261	
Student	0.94 [0.87–0.97]	228		80 [60–85]	194	
Housewife	094 [0.88–1]	258		80 [70–95]	267	
Others**	0.86 [0.66–0.94]	164		60 [50–95]	196	
*Monthly income*						
< 1700 ETB	0.94 [0.85–0.99]	225	0.001*	80 [65–90]	218	0.007*
1700–5000 ETB	0.94 [0.87–0.97]	233		80 [70–90]	241	
> 5000 ETB	0.97 [0.77–0.97]	299		90 [80–96.5]	293	
*Smoking status*						
Daily	0.99	388	0.27	–	293	0.49
Occasionally	0.9 [0.71–1]	215		77.5 [55–92.5]	210	
Not at all	0.94 [0.87–0.94]	257		80 [70–90]	257	
*Alcohol consumption*						
Never	0.94 [0.88–1]	258	0.77	80 [70–95]	260	0.63
Monthly or less	0.94 [0.86–0.97]	242		77.5 [70–90]	237	
2–4 times a month	0.97 [0.8–1]	268		85 [70–90]	256	
2–3 times a week	0.97 [0.88–1]	275		75	271	
*Disclosure status*						
Yes	0. 94 [0.87–1]	257	0.09	80 [70–90]	257	0.35
No	0.88 [0.67–1]	175		75 [70–90]	211	

TVET, Technical and Vocational Education and Training

*Significant value

**Others: Seasonal and unemployed

Kruskal–Wallis analysis indicated that there was a statistically significant difference in EQ-5D utility values among patients with different CD4 count intervals, *p* < 0.001. The number of medicines and comorbidities were significantly associated with EQ-5D utility index and EQ-VAS score, *p* < 0.001. Participants with CD4 range of above 500 cells/mm^3^ had higher (median = 1; IQR = 0.94, 1) EQ-5D utility value while those with CD4 range of below 100 cells/mm^3^ had lower median value (0.88; IQR = 0.66, 0.96). The median EQ-5D utility index value for patients who were receiving more than four medicines at a time was 0.89 (IQR = 0.76, 0.97) while patients on a single medicine had a median value of 0.96 (IQR = 0.9, 1) (Table 5).

**Table 5:** Clinical characteristics associated with EQ-5D and EQ-VAS score of patients with HIV/AIDS, TASH, Ethiopia, 2019 (n = 511)

Patient character	EQ-5D			EQ-VAS		
	Median [IQR]	Mean rank	*p* value	Median [IQR]	Mean rank	*p* value
*CD4 cells/mm*^*3*^						
> 500	1 [0.94–1]	339	< 0.001*	90 [82–99.75]	346	< 0.001*
[350–500]	0.94 [0.88–1]	258		85 [70.25–90]	268	
[200–350]	0.94 [0.86–0.97]	232		77.5 [65–85]	213	
[100–200]	0.88 [0.76–0.95]	172		70 [60–85]	184	
< 100	0.88 [0.66–0.96]	184		60 [50–83.75]	166	
*WHO clinical stage at diagnosis*						
I	0.94 [0.85–0.97]	233	0.06	85 [72.5–90]	264	0.08
II	0.96 [0.87–1]	261		85 [70–95]	266	
III	0.96 [0.89–1]	273		82.5 [70–95]	265	
IV	0.94 [0.86–1]	235		80 [65–90]	229	
*Viral load*						
< 50 copies/ml	0.95 [0.88–1]	263	0.005*	85 [70–95]	267	< 0.001*
51–1000 copies/ml	0.94 [0.88–1]	257		82.5 [70–95]	260	
> 1000 copies/ml	0.88 [0.7–0.98]	196		70 [60–80]	169	
*Type of medicine*						
AZT/3TC/EFV	0.97 [0.94–1]	309	0.004*	82.5 [70–91.75]	286	0.002*
AZT/3TC/NVP	0.96 [0.88–1]	271		85 [71.25–90]	278	
TDF/3TC/EFV	0.96 [0.88–1]	264		85 [70–95]	268	
TDF/3TC/NVP	0.94 [0.89–1]	269		85 [75–95]	280	
ABC + 3TC + EFV	0.88 [0.82–0.96]	189		70 [60–80]	192	
TDF + 3TC + ATV/r	0.82 [0.77–0.97]	253		70 [51.25–85]	238	
Others**	0.94 [0.86–1]	205		80 [65–90]	193	
*Number of medicine*						
1	0.96 [0.9–1]	289	< 0.001*	90 [70–95]	289	< 0.001*
2	0.94 [0.85–0.97]	240		80 [70–90]	246	
3	0.94 [0.86–1]	239		80 [65–90]	240	
4	0.91 [0.83–0.97]	225		75 [60–90]	214	
> 4	0.89 [0.76–0.97]	179		70 [50–80]	144	
*Duration of treatment*						
< 1 year	0.96 [0.94–0.96]	49	0.08	85 [72.5–90]	51	0.12
1–5 years	0.92 [0.86–0.97]	50		80 [66.25–90]	45	
> 5 years	0.96 [0.88–1]	51		82.5 [70–95]	55	
*Adherence*						
Good	0.95 [0.88–1]	259	0.14	80 [70–90]	259	0.08
Fair	0.93 [0.84–1]	225		72.5 [56–93.75]	223	
Poor	0.89 [0.7–1]	209		70 [60–90]	201	
*History of treatment failure*						
Yes	0.92 [0.83–0.97]	210	0.001*	70 [60–85]	195	< 0.001*
No	0.96 [0.88–1]	266		85 [70–95]	270	
*Comorbidities*						
No	0.96 [0.88–1]	216	0.001*	85 [70–95]	213	< 0.001*
OIs	0.92 [0.84–0.97]	273		70 [55–90]	274	
Non-OIs	0.92 [0.8–0.97]	336		80 [65–90]	237	

*Significant value

**Others: ABC + 3TC + NVP, TDF + 3TC + LPV/r and AZT + 3TC + LPV/r; NVP: Nevirapine; ABC: Abacavir; OIs: Opportunistic infect

## Discussion

This study provided critical information on HRQoL, health state utility values and factors that influence the values in the context of HIV/AIDS patients in Ethiopia. The study observed a higher frequency of health problems in all five dimensions of EQ-5D among HIV/AIDS patients compared to the general population of Ethiopia [23]. Consistent with previous studies from different parts of the world, HIV/AIDS patients had higher frequency of problems on the anxiety/depression dimension than the general population [24, 25]. This indicates that patients with HIV/AIDS need better health care services than currently provided to reduce their health problems and improve quality of life with particular focus on mental and palliative care.

Based on respondents’ descriptive profile, higher proportions (45, 71.4%) of the younger population claimed anxiety/depression problem than older age groups. This finding is consistent with a previous study that reported reduced depression symptoms with increase in age [26]. Depression has an impact beyond the reduction in quality of life and it is significantly associated with a fall in the level of treatment adherence [27]. Consequently, the younger population needs more psychological support including HIV-specialist counselling, psychotherapies and mental health intervention to cope with anxiety and depression. These interventions are expected to improve the patient quality of life and to enhance treatment outcomes through improved adherence to the treatment regimen [27, 28]. Almost three-fourth of participants’ older than 60 years of age reported problems on the pain/discomfort dimension. This could be associated with age and comorbidities, showing the importance of managing comorbidities and provision of palliative care for older HIV/AIDS patients [29].

In the current study, the overall median EQ-5D utility index and EQ-VAS score were reported as 0.94 (IQR = 0.87, 1) and 80% (IQR = 70, 90), respectively. The EQ-5D index recorded from this study were higher than the finding reported from Vietnam (mean = 0.65; SD = 0.27) and Colombia (mean = 0.85; SD = 0.21) [24, 30]. The utility value reported from the patients or society is country specific and it can be affected by cultural belief. As a result, such discrepancies usually occur in the utility scores reported from different countries [31]. Since utility is affected by socio-cultural vaue of the society, the higher utility report of the patients in our study may not reflect the better performance of the health care service in Ethiopia. The EQ-VAS scores were recorded as lower than the general population that reported the median value of 90% (IQR = 20) and this could be acceptable since several studies indicated that HIV/AIDS patients have reduced quality of life in comparison to general population [19, 23, 32, 33]. Findings from South Africa and Colombia depicted better EQ-VAS scores in comparison to our study [30, 34]. If we consider the better economic status of South Africa and Colombia the reported utility value from Ethiopian patient seems worthy. But, further work has to be done to improve the HRQoL of the patients with the fact that reported HRQoL of the patients is lower than the general population.

This study examined the difference in utility value of patients based on demographic and clinical characteristics. Patients with low income demonstrated a lower utility value in comparison to the higher-income patient population. Studies showed that HIV/AIDS patients and other chronic diseases showed better HRQoL with improved economic status [24, 35]. HIV/AIDS also affects patients’ economic status keeping individuals and families in the poverty trap [36]. Improved health outcome needs health care services and economic intervention, therefore, providing social and financial support for low-income HIV/AIDS patients should be considered. From our study, it was found that age, occupational status, and household monthly income were significantly associated with the EQ-5D index and EQ-VAS score. This revealed the convergent validity of EQ-5D-5L and EQ-VAS as depicted in a previous study from Vietnam [24].

Participants with a CD4 range of above 500 reported higher utility scores while those with a CD4 range of below 200 claimed lower utility scores. This result is consistent with studies that reported better quality of life with increased CD4 [37, 38]. In addition to the CD4 range, the number of medicines and comorbidities were significantly associated with utility scores. Viral load and history of treatment failure were also found to be associated with EQ-5D utility index and EQ-VAS score of the patient. Existing literature showed that change in medicine and an increase in viral load may cause psychological distress on patients and this would affect the utility score [39]. Therefore, counseling patients on treatment adherence to prevent treatment failure and promoting health education for reducing the magnitude of comorbidities may be helpful in improving their HRQoL.

As a limitation, the study conducted at single hospital and participants for the study were recruited using convenience sampling approach and this may lead to sampling biases. These issues might have influenced the generalizability of the findings. This study has several stregths and contribution to the current literature. To the best of the authors’ knowledge, this study is the first of its kind to establish the utility value for patients with different health states of HIV/AIDS in the Ethiopian setting. The utility value reported from the current study can be used as an input for conducting future economic evaluations of different HIV/AIDS interventions. The study enrolled large sample size and this increases the precision of the finding.

## Conclusion

This study found that anxiety/depression and pain/discomfort were more frequently reported health problems among HIV/AIDS patients. The higher frequency of problems in each health dimensions and lower overall utility values were recorded from HIV/AIDS patients in comparison to the general population. The utility value of the patients was significantly associated with age, income, CD4, the number of medicine the patient was taking at a time and comorbidities. Service providers should work to reduce problems of anxiety/depression and pain/discomfort to further improve patients’ overall utility values.

## Data Availability

The datasets used during the current study are available from the corresponding author on reasonable request.

## Abbreviations

ART: Antiretroviral Treatment
CD4: Cluster of differentiation 4
EQ-5D-5L: EuroQol-5 Dimensions-5 Levels
EQ-VAS: EuroQol-Visual Analog Scales
HIV/AIDS: Human Immunodeficiency Virus/Acquired Immune-Deficiency Syndrome
HRQoL: Health-related Quality of Life
IQR: Interquartile Range
TASH: Tikur Anbessa Specialized Hospital
